# Observational trial of the preventive impact of a social risk factor focused program for co-occurring opioid and methamphetamine use disorders and suicide risk in parents: study protocol

**DOI:** 10.1186/s13722-026-00667-1

**Published:** 2026-04-24

**Authors:** Lisa Saldana, Gracelyn Cruden, Jason E. Chapman, Ryan R. Singh, Jamie Jaramillo, Ally Dir, Mark Campbell, Jeffrey M. Peterson, Maria Bybee, John Radich, Kaylee Becker, Megan Spain, Holle Schaper, Joseph E. Glass

**Affiliations:** 1 Chestnut Health Systems, Lighthouse Institute, 1255 Pearl St., Eugene, OR 97401 USA; 2 Department of Psychiatry, Indiana University School of Medicine, Indianapolis, IN USA; 3 Retired ODHS District Manager, Eugene, OR USA

**Keywords:** Social risk factors, Opioid, Methamphetamine, Child welfare, Prevention, Evidence-based

## Abstract

**Background:**

Parents who experience adverse social risk factors of health (SRFOH) are at risk for child welfare involvement, and those with opioid and/or methamphetamine use (O/MU) and mental health (MH) disorders are at risk for escalating negative outcomes like intravenous (IV) drug use and suicide. Just Care for Families® is a continuum of preventive care program that targets parental SRFOH, including those with O/MU and MH disorders. This protocol describes an observational trial of Just Care, spanning five rural counties that will examine malleable individual SRFOH (e.g., employment) and systemic SRFOH (e.g., access to quality healthcare), and less malleable structural SRFOH (e.g., county-based health risk factors), along with the program’s impact on outcomes.

**Methods:**

Parents referred to Just Care for Families (*N* = 250) across five rural Oregon counties will be recruited. County health metrics will be collected from the areas where participants live to examine structural SRFOH. Consenting parents will report their SRFOH needs weekly for 18 months, regardless of engagement in Just Care services. When engaged, Just Care coaches will report weekly on whether social risk factors were identified, intervened upon, or successfully addressed. Parents will be assessed for O/MU, including IV drug use, and MH symptoms, including suicide (ideation, intention, attempt) at Baseline, 9- and 18-months. Administrative health data will be collected from consent to 24–42 months post-Baseline to assess longer-term prevention of IV drug use and suicide. Cost data will focus on costs to deliver the intervention and costs reimbursed to clinics. An intensive, longitudinal, sequencing design will allow an analysis of how intervention components disrupt malleable individual SRFOH, malleable systemic SRFOH, and escalation of O/MU and MH disorders (Aim 1); how intervention effects are impacted by less malleable structural SRFOH (Aim 2); and how the relationship between SRFOH and both individual and system outcomes influences costs incurred by provider clinics delivering Just Care for Families (Aim 3).

**Discussion:**

Outcomes will inform program scale-up by providing an empirical basis for targeting parent SRFOH throughout the course of treatment, as well as the impact of these clinical decisions on parent outcomes and clinic-borne costs.

**Trial registration:**

NCT06560866 registered and released 7/10/2024.

## Background

The social risk factors of health (SRFOH) shared between parental opioid and/or methamphetamine use (O/MU), mental health (MH) disorders, child maltreatment, and subsequent child welfare system (CWS) involvement—as well as the cyclical and transgenerational nature of these relationships—are well-known [1–3]. Less understood is how to target these SRFOH and disrupt their negative effects. A multi-component, multi-level preventive intervention could avert cascading negative individual and interpersonal outcomes for parents and their families. The current study seeks to test this type of intervention.

### The impact of SRFOH on Families with O/MU and MH disorders

Families with parental O/MU and MH disorders have a high likelihood of poor SRFOH in all five areas identified by Healthy People 2030 as essential for healthy development and quality of life: economic stability, access to quality education, healthcare, neighborhood and built environment, and social and community context [4, 5]. For example, individuals who present with O/MU disorders often have low income status, un- or underemployment, limited access to healthcare and education, and housing difficulties [6, 7]. For individuals experiencing housing instability, the O/MU overdose rate has increased dramatically in recent years [7]. Notable to this study is the bidirectional nature of SRFOH, and O/MU and MH disorders [8]. Moreover, the children of parents with O/MU and MH disorders are at increased risk for exposure to negative SRFOH and developing poor health trajectories of their own.

### Study context: an identified urgent need

Throughout the United States, there are strong interrelationships among O/MU, MH disorders, and poor SRFOH. In states like Oregon, this comorbid presentation increased during the COVID-19 pandemic [9, 10]. Even prior, Oregon struggled to meet the need for addiction and MH services throughout the state, resulting in the governor declaring a statewide crisis in 2018 (HB 4134; HB 4137).

Since this declaration, a continued alarming rise in O/MU throughout the state has created a watershed moment. In January 2022, data were released [11, 12] ranking Oregon second nationally for substance use disorders—with 18.22% of the population experiencing addiction to substances—and first for addiction to illicit drugs (9.04%). Oregon still ranked first in opioid prescription misuse and evinced a 53% increase in methamphetamine use (i.e., 23,000 newly identified individuals). This notoriety was accompanied by a simultaneous decline in the national ranking to 50^th^ for access to treatment.[12]

These national data also ranked Oregon second for MH disorders and third for severe mental illness, with 27.33% and 7.15% of the respective populations diagnosed in the last 12 months. Oregonians ranked second for suicidal ideation (6.80% of the population) and fifth for experiencing a major depressive episode within the last year (9.84%) [12]. Data indicated that 30.8% of fatal overdoses in Oregon were experienced by individuals with a diagnosed MH disorder; yet, only 8.7% were receiving treatment for either MH or substance use disorders [13].

In 2020, there were 78,632 referrals to Oregon’s CWS, 10% of which were substantiated and resulted in the placement of 9,838 children into foster care for at least one day. Referrals most often were for parental drug use (41%), followed by domestic violence (32%), which often is associated with substance use. The rates of substantiated child maltreatment cases were highest in rural counties [14]. Rural conditions present unique challenges (e.g., limited public transportation, few providers) for disrupting the effects of adverse SRFOH, which is particularly important given the high prevalence of O/MU and MH disorders in these regions [9, 15–17]. In 2020, while unknown how many individuals who overdosed were actively parenting, the risk of fatal overdose was higher for individuals living in rural versus urban Oregon counties, and was 14% higher for those who were unhoused compared to those who were housed [13]. Nationally in 2020, 45% of all overdose-related deaths were related to intravenous (IV) drug use [18] and in Oregon, one survey found 88% of individuals who used drugs reporting IV-drug use [19].

Thus, Oregon’s O/MU and MH crises provide an urgent and unique opportunity to examine the impact of intervention on parental SRFOH, SU, and MH for preventing IV drug use and suicide (ideation, intention, or attempt). The current study will examine Just Care for Families®—an intervention addressing the complex needs of parents involved in CWS, including substance use and MH treatment, parent skills training, community building, systems navigation, and meeting basic needs. Just Care for Families directly targets individual and systemic SRFOH, aiming to disrupt their trajectory to improve outcomes related to O/MU and MH disorders.

### Intervention: Just Care for Families

Just Care for Families is a multicomponent clinical intervention designed to provide integrated, evidence-based treatment strategies to address the shared correlates and intersection of substance use, MH, and parenting needs, along with a treatment plan focused on building community, navigating systems, and addressing basic needs. Using a well-specified behavioral approach, Just Care for Families treatment is individualized to fit the unique circumstances and needs of participating families with parental O/MU and MH disorders. Just Care for Families comprises six treatment components each of which are individualized to meet parents’ needs: (1) substance use treatment including contingency management and positive reinforcement; (2) MH treatment including behavioral activation, developing healthy coping skills, emotion regulation skills, exposure therapy, and referral for medication management; (3) parent skills training focused on nurturing and attachment, reinforcement, and meeting child SRFOH needs; (4) community building focused on developing prosocial connections; (5) systems navigation including CWS, probation, housing system, adult and/or child education system, and other social service systems, and (6) meeting basic needs including food security, identifying safe and stable housing, documentation (e.g., birth certificate, social security card, immunization records), identifying safe childcare, and physical safety. Program-specific behavioral assessment tools are used throughout treatment to help coaches and parents work together to understand the reasons behind parent behaviors and symptoms and to in turn, reinforce positive behavioral changes.

Trauma informed treatment components are delivered by a designated Just Care Coach working at a community behavioral health clinic offering Just Care for Families. Coaches are dually certified as certified alcohol and drug counselors and qualified mental health associates and are supervised by a master’s-level supervisor. All treatment is delivered in community- or home-based settings (e.g., home, school, playground) so that parents have the opportunity to practice intervention strategies regardless of their living environment (e.g., house, camp, park). The Just Care for Families coaching team is available 24/7 for on-call support and ongoing engagement. The team collaborates closely with CWS staff to help parents align treatment goals with those needed to accomplish their CWS goals.

Just Care for Families starts out more intensely, with sessions titrating down over the approximately 9-month course of intervention. In the first three weeks of treatment, parents meet daily with their Coach, provide an instant urinalysis, and participate in a collaborative team meeting with their child welfare caseworker and Coach. As parents achieve sobriety and greater mental health and SRFOH stability, session frequency decreases. Session lengths vary from a short 15-minute check-in and urinalysis to several hours. Sessions can increase or decrease in both length and frequency as needed throughout Just Care for Families to accommodate each parent’s individual treatment needs. Parents are eligible for graduation once treatment goals have been met and they have obtained a minimum of 90-days free from the substances being treated. Warm handoffs for ongoing follow-up with mental health or substance use providers are made if indicated.

#### Addressing individual and systemic SRFOH

Just Care for Families specifically assesses, identifies, and targets SRFOH that frequently arise for referred parents (e.g., unemployment, lack of transportation, food insecurity, exposure to violence, and discrimination). Intervention components range from basic goal setting to helping parents act on and accomplish those goals (e.g., submit employment applications, establish care with a medical provider). Not all parents have the same SRFOH needs, and not all Coaches have the same access to resources for each parent on their caseload. Thus, although addressing individual SRFOH needs is a treatment priority, the solutions are not scripted, allowing for variability between cases in how and when SRFOH are addressed. Just Care for Families thus presents a unique opportunity to examine patterns of influence that disrupt individual SRFOH and prevent subsequent negative O/MU and MH outcomes.

Just Care for Families identifies and targets SRFOH for parents in the context of other systems, including access to equitable primary care, legal representation, and support from the CWS and criminal legal system. Coaches support parents when interacting with other service providers and provide skills training to interact with and navigate service systems. Similarly, when participating in service provider partner meetings, Coaches model for other providers positive ways of interacting with parents and engage in coordinated case planning that emphasizes parents’ strengths and positive progress—all with the goal of improving service provider-parent interactions and the subsequent quality of care received.

### Hypothesis 1: Just Care for Families will reduce individual and system-level SRFOH and escalation of O/MU and MH disorders

**Fig. 1:**
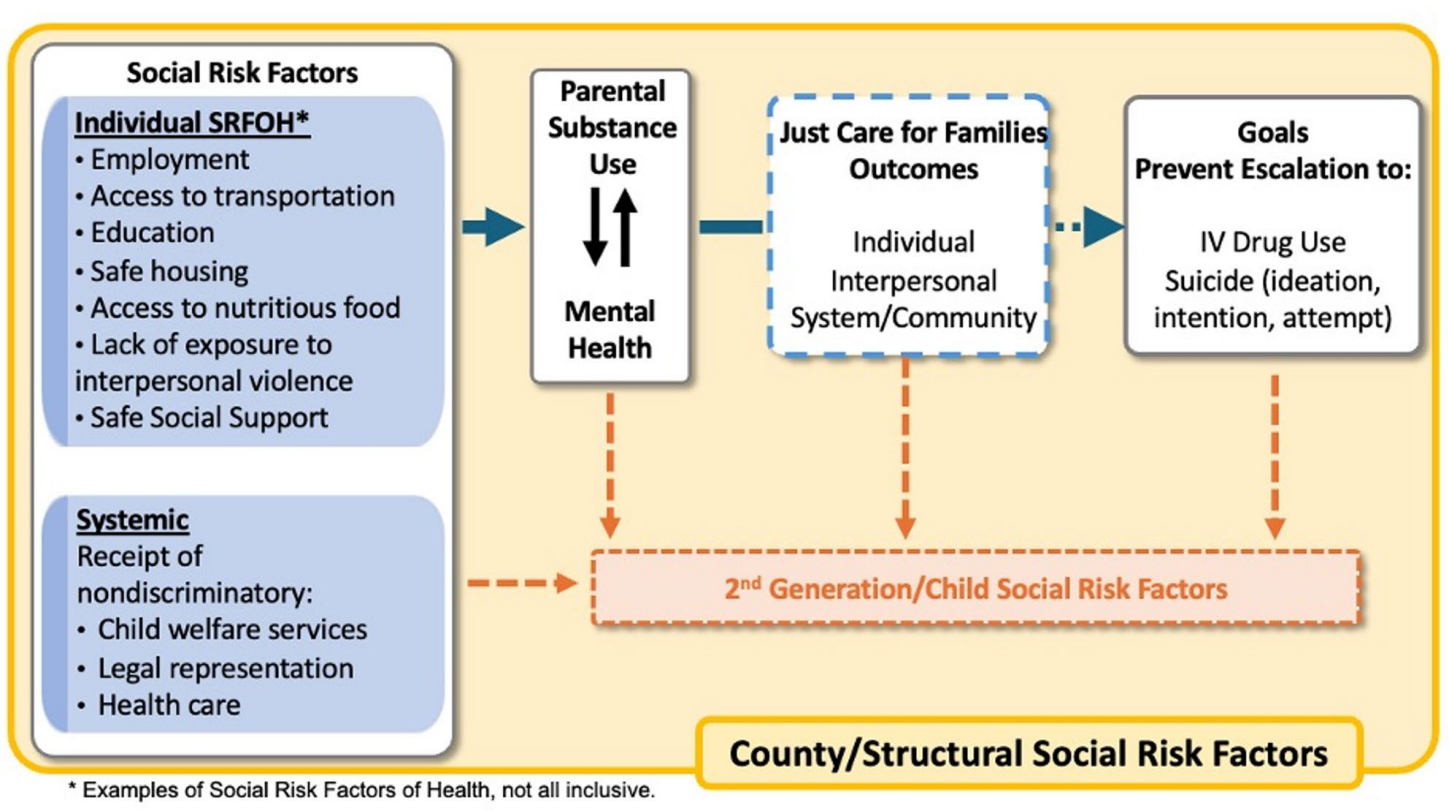
Study logic model. Endorsement or not of social risk factors is hypothesized to influence parental substance use and mental health. Just Care for Families® is expected to disrupt the negative path for parents and prevent parental escalation of drug use and mental health symptoms. Study outcomes are expected to influence distal (not measured) second-generation social risk. factors

This study tests the hypothesis that Just Care for Families components target the individual and systemic SRFOH that drive parental O/MU and MH disorders, and subsequently improve outcomes at the individual, interpersonal, and community/system levels. The program addresses a range of malleable individual and systemic SRFOH. Each of these risk factors is positioned within a broader structure of less malleable risk factors, including ruralness and geographic region, and their associated factors (e.g., access to health care, housing). The overarching premise of this study (Fig. 1) is that by targeting the malleable individual and systemic SRFOH that drive the bi-directional relationship between parental O/MU and MH disorders and subsequent worsening outcomes, Just Care for Families can disrupt the negative trajectory of individual, interpersonal, and system/community intermediary outcomes, preventing transition to IV drug use or suicide (ideation, intention, or attempt).

#### Objective: Aim 1

Intensive longitudinal data will be explored using time-series analyses to evaluate the mechanisms of action for the Just Care for Families program components on the ultimate prevention outcomes of transition to IV O/MU and suicide (ideation, intention, attempt). The two types of mechanisms include improvement in (1) individual and system-level SRFOH (the direct targets) and (2) substance use and MH symptoms (intermediate prevention outcomes).

### Hypothesis 2: SRFOH that are addressed in Just Care for Families are impacted by external infrastructure factors

Although parents might present with a SRFOH that is identified as a significant driver for O/MU and MH symptoms, some risk factors are not directly within the control of the individual (e.g., lack of public transportation, local access to a prescriber). It is hypothesized that Just Care for Families treatment goals and subsequent effects will be impacted by the ease with which the Coach can help the parent address the identified need.

#### Objective: Aim 2

Moderation analyses will evaluate county-level risk factors—including community education, poverty, housing, transportation, and healthcare service availability—as potential moderators of the associations across delivery of Just Care for Families program components, individual SRFOH, system-level SRFOH, intermediate prevention, and ultimate prevention outcomes.

### Hypothesis 3: Delivery of Just Care for Families can influence the relationship between SRFOH and individual/system-level outcomes on clinic-borne cost

This study tests the hypothesis that the impact of Just Care for Families on clinical outcomes (preventing escalation of symptoms to IV drug use or suicide) will have a subsequent impact on clinic operations, including caseload turnover and reimbursement. The transition of parents to IV-drug use or suicidal behaviors is proposed to significantly impact the course of treatment, movement to treatment graduation, and subsequent long-term outcomes on child removal into foster care. Individual parents present with different levels of needs and associated treatment plans. As parent SRFOH needs are addressed, their treatment trajectory is proposed to improve, impacting the intensity of treatment needs (e.g., frequency of contact, number of in-person versus remote sessions) and length of time in treatment (e.g., shorter or longer time between initiation and termination). These changes in SRFOH needs, in turn, impact program delivery expenses such as non-reimbursable drive-time (i.e., to meet parents in the community), and ability to turn over caseloads and meet waitlist demands. These operational outcomes impact clinic-borne costs. It is hypothesized that long term, Just Care for Families’ ability to prevent escalation to IV drug use or suicide also will impact CWS costs.

#### Objective

Using system dynamics, the average total cost to deliver Just Care for Families per parent who received it will be evaluated. Specific to the provider perspective, clinic-borne costs (the difference between reimbursed and actual costs, including fixed costs and unbillable hours) will be evaluated. Costs also will be examined for parent profiles, defined by which SRFOH needs are addressed and the order in which they are addressed.

### Study opportunity

This protocol leverages Just Care for Families programs that are operating in five rural Oregon counties. County variation in SRFOH will allow the opportunity to observe the moderating effect of structural SRFOH on individual and system outcomes. There is a unique opportunity to evaluate the disruption of malleable SRFOH as a mechanism for prevention of initiation of IV drug use and suicide (ideation, intention, and attempt). These effects are expected to vary based on less malleable county-level indicators of SRFOH. The design includes intensive measurement of the Coach’s delivery of specific intervention components for each SRFOH, and this is documented each session for each parent throughout the course of treatment.

## Method

### Community advisory board

A community advisory board (CAB) serves as paid consultants to the investigative team (when allowed). The CAB will meet annually (in-person) and twice a year virtually. The CAB will comprise a parent representative from each county (*N* = 5), a clinical supervisor from each clinic (*N* = 4), a representative from the Oregon Department of Human Services (ODHS) fatality board (leading efforts to address risk for suicide and substance use), and community partners (e.g., representing temporary housing/shelter, peer recovery services, tribal services) addressing SRFOH at the community level. The CAB is co-led by a retired ODHS District Manager and an early-career co-investigator. Input was sought on measures, intervention targets, and will be sought on the interpretation of outcomes, and resulting conclusions that are shared with interested systems. CAB meetings will inform Aim 3 system dynamics modeling. The CAB will (1) serve an essential role in providing input into the bidirectional association between SRFOH and O/MU and MH outcomes, and (2) provide advice to the investigative team regarding how individual and structural contextual factors might impact outcomes.

### Overview of study procedures

Institutional Review Board approval was obtained for all participant recruitment procedures, consents, and releases of information. This study is a prospective single-group intervention study that harnesses the real-world variability in the delivery of Just Care for Families, alongside comprehensive longitudinal assessments, to examine SRFOH, parent outcomes, and associated mechanisms. Interventions are not manipulated by study procedures; instead, they will be observed as they are delivered, with hypothesized reductions in participant symptoms over time. Study data are from multiple data sources: comprehensive assessments of parent SRFOH, O/MU, MH symptoms, and family functioning (Baseline, 9-months, 18-months); weekly digital check-ins of parent self-reported SRFOH needs; weekly Coach reports of parent-specific SRFOH priorities and interventions; administrative data collected from ODHS and Medicaid; and publicly available data on county structural SRFOH metrics. Measures collected as part of the study research procedures are not included in clinical intervention, and responses are not shared with the clinical team.

### Recruitment

#### Just Care for Families clinical team members

All Just Care for Families teams operating in the five participating counties have agreed to participate in the study. Coaches will be recruited to participate in clinical data collection and will be compensated $20 per week for their time spent on timely documentation of SRFOH activity for each participating parent. Coaches will not be asked to change anything about their clinical practice for the study, only to record what is done. Coaches (*n* » 41) will be eligible if they (1) are in a participating clinic, and 2) provide Just Care for Families services to parents at any point in the study.

#### Parents

Two hundred and fifty parents will be recruited. Referrals will be made to the project recruiter from CWS case workers, other community partners, and parents themselves. The project recruiter will conduct a phone screen with the parent to determine study eligibility: (1) Use or misuse of opioids in the last year, and/or any methamphetamine use, and/or other non-prescribed drug use in the last year, (2) parent of a child, age 0–18, residing in the home or with a reunification plan in place (3) MH symptoms, (4) residing in one of the five participating counties, (5) insured by Medicaid, (6) access to a computer, smartphone, or wireless/cellular connection if a device were to be provided, or reliable access to a landline to receive a brief weekly phone call in place of digital check-in.

Eligible parents will be scheduled for an initial baseline assessment at times and places that are most convenient for parents. The assessor will describe the study, review the IRB-approved informed consents, and obtain parental consent. Parents will be compensated with a gift card of their choice for participating in both major assessments ($100) and weekly surveys ($10) immediately upon completion.

### Participant tracking

Participating parents will be followed individually for 18 months and administratively for 24–42 months post-baseline, depending on the enrollment date. Assessors will be assigned to individual participants to help establish long-term rapport and ensure retention. Assessors will begin reaching out to participants one month before their assessment due date to schedule follow-up assessments. In addition to conducting assessments at times and places that are convenient for the participant, assessors will use other engagement strategies, such as providing a favorite beverage or snack at the time of major assessments (i.e., baseline, 9- and 18-months). To overcome parent disengagement, assessors will drive by the last known residence, use alternative contacts provided at baseline with a release of information, and provide small incentives for parents who contact the team and provide updated contact information. Parents will be located using social media (through a Just Care for Families program account) and web-based searches (e.g., People Search, public records). Parents can opt out of research participation at any time without any consequence to their Just Care for Families treatment or child welfare case.

### Major assessment procedures

Multi-instrument psychosocial assessments and urine drug screens will be completed at baseline, 9 months, and 18 months. The baseline assessment will occur at the time of consent, except in instances in which a participant is placed on a waitlist following referral to Just Care for Families. If waitlisted, the baseline assessment will be delayed until notified by the clinic that the participant has been scheduled for intake. To coordinate the timing of the baseline assessment, the project screener will maintain communication with clinic supervisors in counties offering Just Care for Families to determine if there is a waitlist for treatment. The subsequent 9-month and 18-month assessments will be anchored from the date of the baseline assessment. Assessments will occur at times and places that are most convenient for parents.

Major assessment protocols aim to be 2 hours or less, including breaks. Major assessments will be conducted in person or using HIPAA-compliant virtual technology. Assessors live within participating counties and have been hired and trained to provide in-person contacts and conduct participant tracking. All assessments will be administered using REDCap. Paper and pencil will be available as a backup or, if preferred by the parent.

Protocols are in place for participant endorsement of “red flag” items, including suicidal or homicidal ideation, or identification of a participant’s parenting while under the influence of substances. When these items are endorsed, the assessors will contact the assessment supervisor while remaining with the participant. The supervisor will contact the Qualified Mental Health Professional on the team to conduct a risk assessment. If a participant is screened as high-risk or at imminent risk of harm to self or others, the study Principal Investigator (a clinical psychologist) will be contacted for additional assessment and intervention. At a minimum, all individuals who endorse red flag items will be provided information for local resources. Individuals who are assessed with higher needs will be assisted in contacting their Coach or other preferred service provider. If imminent risk is determined, appropriate authorities (i.e., law enforcement, mobile crisis, child welfare) will be contacted. Prior research by the investigative team, with similar samples, suggests a relatively high rate of red flag endorsements (e.g., UH3DA050193; 28% of assessments were flagged, 61.5% of which were flagged for “*thoughts of death or dying”*).

### Major assessment measures

#### Demographics questionnaire

The demographics questionnaire includes items provided in the PhenX toolkit [20] race, ethnicity, gender identity, sex, age, and family constellation.

#### Lifetime social risk factors of health

Individual/interpersonal SRFOH will be assessed using measures identified through the PhenX toolkit [20], including access to health services (e.g., insurance, physical, mental health, dental) [21], annual family income [22], current employment status [23], educational attainment [24], language proficiency [25], food insecurity [26], health literacy [27], and discrimination [28]. In addition, interviews will assess whether the participants have experienced: being unhoused; CWS or juvenile justice involvement as a child; having an incarcerated parent; family violence; and social isolation. The baseline interview assesses lifetime social risk factors, and these risk factors will be reassessed at follow-up interviews. Additional social risk factors of health will be assessed in weekly surveys (see below).

#### The Addiction Severity Index (ASI) [29]

The ASI is a standardized tool for evaluating the type, frequency, and amount of substances used, including route of administration, and thus, will be used to measure the primary outcome of interest of past 30-day IV drug use. In addition, it is a rich source of data regarding SRFOH and correlates of substance use, including family history, history of physical, sexual, and verbal abuse, housing stability, legal system involvement, education attainment, and employment status. This self-report assessment includes behaviors and experiences across the lifespan and over the last 30 days. The ASI has strong psychometrics and is part of the PhenX toolkit [20].

#### Urine drug screens (Confirm BioSciences)

12-panel urine toxicology plus fentanyl screens will be collected at each major assessment. Participants will be reminded that participation is voluntary. Parents who have custody of their children also will be reminded that a positive screen for non-prescribed illicit drug use might lead to a report to child welfare. CLIA-waived Instacups will be used for collection and include temperature and adulterant tests as part of the kit. All collections will be observed (client preference for observer sex is accommodated through assessor assignments), with refusals recorded as such and coded as positive/refused. In the case of a positive screen, all substances that are positive are recorded. Participants will be asked to provide prescriptions (e.g., bottle, pharmacy note) for prescribed medications (e.g., buprenorphine), and these positive screens will be recorded accordingly. Urine Drug Screens that are recorded as positive/refused or positive/prescribed will not lead to a child welfare report. As is done in prior studies, a picture will be taken of the testing strip for data entry validation.

#### The Brief Symptom Inventory (BSI) [30]

includes 53 self-report items that assess psychiatric symptoms on nine subscales, including depression and suicidality. It has strong convergent and predictive validity.

#### Life Events Checklist for DSM-V (LEC) [31]

Selected from the PhenX toolkit [20], the LEC is a 17-item list of stressful or potentially traumatic experiences to which an individual might have been exposed. Individuals select whether the event ever happened to them, they witnessed it, learned about it, experienced it as part of their job, or if unsure/does not apply. The LEC yields adequate psychometric properties.

#### Trauma Symptom Checklist (TSC) [32]

The 40-item TSC assesses how often an individual has experienced each symptom in the last two months on a 4-point scale (ranging from Never to Often). Total scores range from 0 to 120 and produce six subscales.

#### Patient Health Questionnaire (PHQ) [33]

The PHQ is a 9-item self-report measure of depression. The PHQ-9 is a reliable and valid measure of depression severity.

### Weekly assessment procedures and measures

Parents and Coaches will provide weekly independent reports during the course of treatment, yielding intensive longitudinal measurements of SRFOH (parents) and intervention components (Coaches). Unlike major assessments, weekly reports will allow a rigorous assessment of parent SRFOH needs as they arise, if coaches integrate needs as they emerge into treatment planning, and if they are addressed, how quickly the need is addressed.

#### Parent report

At the time of consent (regardless of whether a major baseline assessment has been completed), parents will be enrolled in the REDCap survey app used to collect weekly SRFOH data. Then, parents will complete a brief, weekly digital check-in on the individual and systemic SRFOH status and needs. Responses will not be shared with Coaches. Seven domains of SRFOH will be assessed: work and money (6 items); neighborhood & transportation (4 items); education and training (4 items); food (3 items); community, safety, and support (11 items); healthcare system (8 items); and internet and phone (1 item). In addition, health and wellbeing outcomes (e.g., substance use, mental health) will be assessed weekly as an additional measure of intermediate prevention outcomes.

The weekly assessment will operate through a push notification system in REDCap, which leverages an API with a third-party digital gift card vendor to compensate parents ($10) immediately upon completion of their weekly survey. Each Sunday, parents will receive a prompt to complete their survey. If the parent does not complete the assessment within 2 days of the first prompt, a second will be sent. If no response is received by the end of Saturday, the data will be recorded as missing. A new request will be pushed the following week. If parents do not respond for two weeks of digital check-ins, assessors will engage in additional outreach efforts (e.g., direct text, call, social media message). Digital check-ins will be probed regardless of the parent’s engagement in Just Care for Families and will occur from baseline to 18-months, allowing for a sensitive examination of the relationship between changes in Just Care for Families delivery, SRFOH domains, and health and well-being outcomes (including intermediate outcomes of substance use and mental health).

#### Coach report

As part of standard program delivery, Just Care for Families Coaches will record session information (e.g., session attendance, urinalysis results) in Just Portal – an online session tracker developed for the program. A module was added to include reports of SRFOH targets in each parent’s Just Care for Families weekly treatment goals that mirror the domains of the parent’s weekly self-report. Individual risk factors will be probed to determine if the need has been identified, and if so, how (raised topic, goal setting, action, accomplished). Coach reports will be collected over each participant’s course of treatment (i.e., estimated nine months).

### Administrative data sources

#### Just Care for Families program delivery data

As part of standard compliance and quality assurance procedures for Just Care for Families, group supervision and case discussions are recorded weekly for expert fidelity review and feedback. These recordings will be coded by a team of expert coders (20% recoded for reliability).

#### State administrative data

State administrative data will be collected from ODHS and the Oregon Health Authority (i.e., Medicaid) through separate data sharing agreements with the investigative team. To measure prevention outcomes beyond study participation and to estimate costs for Specific Aim 3, administrative data will be collected for all parents in the final year of the study and will include documented ODHS indicators of family stability (e.g., child removal, child reunification, parent re-report), SRFOH status (e.g., employment, housing), and medical or behavioral health encounters related to O/MU and MH disorders between baseline and administrative retrieval (i.e., baseline to 24–42 months post baseline).

### Public data sources

#### Less malleable Regional/structural SRFOH

Publicly available data will be collected to describe structural SRFOH in the five counties. Measures of community education attainment, concentrated poverty, social vulnerability, housing, and overall health will be collected using PhenX toolkit [20] data sources and protocols. County structural SRFOH also will be gathered in collaboration with ODHS from state available databases.

### Primary, Secondary, and other outcome measures

Data from major assessments and weekly assessments will be used to construct the study outcome measures (see Table 1). Program delivery data from Just Portal will be used to capture intervention dose and session attendance.

**Table 1:** Primary, secondary, and additional outcomes in the Just Care for Families® social risk factors of health (SRFOH) study

Outcome	Definition	Time Frame	Source
**Primary outcomes**			
Opioid use (self-report)	Any non-prescribed opioid use	Past 30 days	Addiction Severity Index
Methamphetamine use (self-report)	Any methamphetamine use	Past 30 days	Addiction Severity Index
Depression with suicidality	Intensity of depression-related distress (Sum of responses to 6 Likert-type items, range 0–24)	Past week	Brief Symptom Inventory Depression Subscale
Anxiety	Intensity of anxiety-related distress (Sum of responses to 6 Likert-type items, range 0–24)	Past week	Brief Symptom Inventory Anxiety Subscale
SRFOH	# of needs or problems across SRFOH domains (# of items endorsed out of 43 items comprising 7 risk factor domains)	Past week	Weekly parent survey
**Secondary outcomes**			
Opioid or methamphetamine use (urine sample)	Positive urine drug screen for opioids or methamphetamines	Current	Urine toxicology plus fentanyl screen
Escalated or continued IV drug use	Presence of IV drug use	Past 30 days	Addiction Severity Index
Suicidal ideation or attempts-1	Presence of any suicidal ideation or attempts	Past 30 days	Addiction Severity Index
Suicidal ideation or attempts-2	Presence of any suicidal ideation or attempts	Past 2 weeks	Patient Health Questionnaire-9 (PHQ-9), ninth item
SRFOH: Specific health and well-being outcomes	Individual needs or problems related to health or wellbeing: overall health, doing everyday activities or responsibilities, parenting, mental health, substance use, physical health (6 separate items)	Past week	Weekly parent survey
**Other Outcome Measures**			
Stressful or traumatic experiences	Exposure to stressful or potentially traumatic experiences (# of items endorsed out of 17 items)	Since the last interview	Life Events Checklist
Depression symptoms	Overall summed score of 9 items	Past 2 weeks	PHQ-9
Days of methamphetamine use	Count of days of methamphetamine use in the past 30 days	Past 30 days	Addiction Severity Index
Trauma symptoms	Frequency of trauma symptoms (Sum of responses to 40 Likert-type items, range 0–120)	Past 2 months	Trauma Symptom Checklist
SRFOH: Work and money	# of needs or problems related to work and money (# of items endorsed out of 6 items)	Past week	Weekly parent survey
SRFOH: Neighborhood and transportation	# of needs or problems related to neighborhood or transportation (# of items endorsed out of 4 items)	Past week	Weekly parent survey
SRFOH: Education and training	# of needs or problems related to education or training (# number of items endorsed out of 4 items)	Past week	Weekly parent survey
SRFOH: Food	# of needs or problems related to food (# number of items endorsed out of 3 items)	Past week	Weekly parent survey
SRFOH: Community, safety, and support	# of needs or problems related to community, safety, and support (# number of items endorsed out of 12 items)	Past week	Weekly parent survey
SRFOH: Healthcare system	# of needs or problems related to healthcare (# number of items endorsed out of 8 items)	Past week	Weekly parent survey
SRFOH: Internet and phone	# of needs or problems related to internet and phone (# number of items endorsed out of 1 item)	Past week	Weekly parent survey

## Data analysis strategy

### Overview of Aim 1 and Aim 2 analyses

**Fig. 2:**
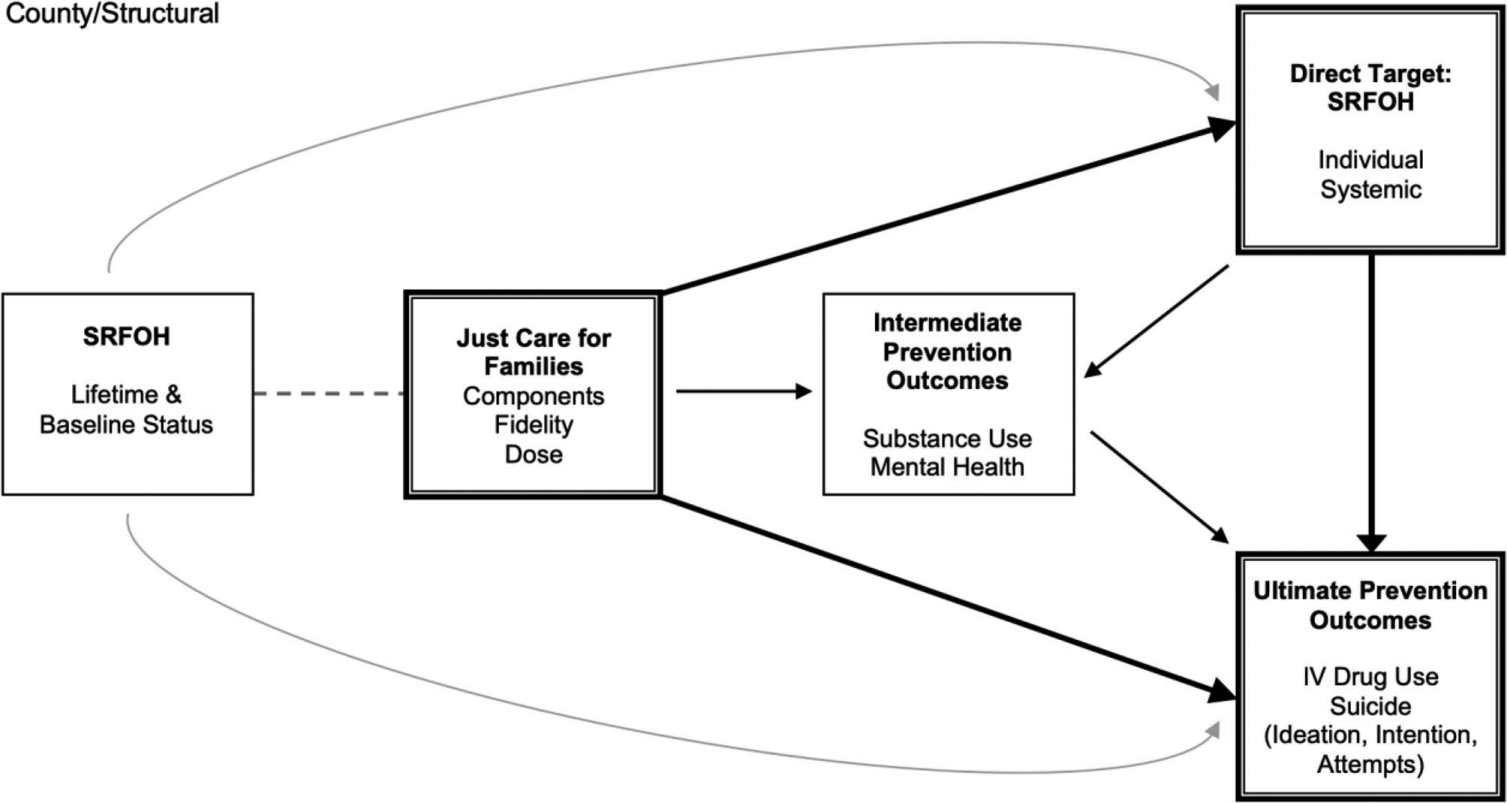
Proposed analytic model hypothesizing two mechanisms of action of the Just Care for Families intervention

The statistical analyses will provide multiple tests of the mechanisms by which Just Care for Families disrupts SRFOH. Ultimate outcomes to be examined are preventing IV drug use and suicide ideation, intention, and attempts. Just Care for Families’ effect on these outcomes is hypothesized to occur through two mechanisms of action: (1) improvement in malleable SRFOH (direct targets of intervention) and (2) improvement in substance use and MH disorders (intermediate prevention outcomes). These mechanisms are evaluated in Aim 1. Aim 2 focuses on whether the effects vary as a function of less malleable external, structural SRFOH, such as community poverty and distance to resources; see Fig. 2.

Two design features have important implications for evaluating the proposed mechanisms of action. First, all participants will be assigned to Just Care for Families, and Coaches provide components of the Just Care for Families treatment “package” as clinically indicated. Analyses will evaluate specific time-varying intervention components (fidelity, dose) and, most importantly, discrete components that target specific SRFOH. Second, to attenuate potential limitations of the non-randomized design, all paths of the proposed mediation models (i.e., XàMàY) will be longitudinal. Final models evaluating sex as a biological variable will examine if intervention effects differ by sex. The analyses articulated below reflect statistical analysis plans at the time of writing this protocol; subsequent changes will be logged and dated in the study’s Statistical Analysis Plan, which will be made publicly available in a supplement to the primary outcomes manuscript.

#### Data structure

Data for Aims 1 and 2 are longitudinal and characterized by nesting, with repeated measurements (level-1) within parents (level-2) within Coaches (level-3). Intervention components (coder- & Coach-reported) and SRFOH targets (parent-reported) are measured weekly throughout treatment. Intermediate and ultimate prevention outcomes (parent-reported) are measured at 0, 9, and 18 months post-baseline; weekly (for health and wellbeing assessments); or based on event occurrence from baseline to 24–42 months (administrative). Coaches are nested within clinics and counties, but with too few for additional random effects, systematic differences will be controlled with dummy-coded indicators. The nested data structures will be addressed using multilevel models in Mplus [34] and other suitable multilevel statistical software, as indicated (e.g., R) [35]. Each variable will be evaluated to confirm the distribution for modeling, with dichotomous and count outcomes modeled with binomial and negative binomial or Poisson distributions.

The primary approach to addressing missing data on outcome variables will be to take advantage of full-information maximum likelihood estimation, which accommodates missing data by using all available data in model estimation. When weekly data are aggregated into longer time periods to coincide with major interviews, the mean score will be calculated across all available weeks, excluding weeks with missing data. If missing data exceeds 10%, the robustness of results will be assessed by estimating models with multiply imputed data with chained equations under a missing at random assumption.

#### Statistical test for mediation/mechanism of action

The statistical tests for mediation will be based on the product of coefficients with asymmetric, bootstrapped *SE*s and 95% CIs for the product [36]. The tests will be performed in Mplus [34] with the corresponding bootstrap and 95% CI specifications. The product of coefficients test requires estimation of two coefficients (a-path & b-path) and their *SE*s. The a-path estimates the effect of intervention components (X) on mediators (M). The b-path, controlling for intervention components (X), estimates the association of mediators (M) on outcomes (Y).

#### Aim 1. Examine how Just Care for Families program components disrupt individual and systemic SRFOH and escalation of O/MU and MH outcomes

The model in Fig. 2 includes two sets of primary outcomes that serve as mechanisms of action—improvements in malleable SRFOH and reductions in substance use and mental health problems. These sets of primary outcomes result in several focal models for analysis: change in malleable SRFOH (direct target) as a mechanism of the effect of Just Care for Families intervention components on (1) intermediate prevention outcomes and (2) ultimate prevention outcomes. In addition, (3) a change in intermediate prevention outcomes is posited as a mechanism for the effect of malleable SRFOH on ultimate prevention outcomes. Analyses involving #1 above will utilize weekly measurements or major interview measurements in separate models, and analyses #2–3 will utilize major interview data in combination with intervention delivery and weekly interview data aggregated into larger time periods to coincide with major interview periods.

#### Mediation analysis involving weekly assessment intervals

A fully longitudinal mediation analysis will be conducted in models examining weekly measurements to evaluate the hypothesis that malleable SRFOH mediates the association between Just Care for Families intervention components and health and well-being outcomes. To address temporal sequencing, our primary analyses of weekly measurements will use lagged measurements (intervention delivery at weeks 1 … 76; malleable SRFOH at weeks 2 … 77; and health and wellbeing outcomes at weeks 3 … 78). Secondary analyses will use concurrent measurements of these constructs (intervention delivery, malleable SRFOH, and health and well-being at weeks 1 … 78).

Analyses will utilize an “unconflated multilevel structural equation” model that decomposes variation between parents and the variation within parents across time [37]. Decomposing the “within” and “between” variance components allows the pathways of the mediation analysis to explicitly model changes within parents over time. This approach to effects decomposition, in combination with temporally sequenced measurements, will allow for a highly rigorous mediation analysis. As stated above, each weekly measurement of intervention components will predict the subsequent week’s malleable SRFOH, and in turn, each malleable SRFOH will predict the subsequent week’s health and wellbeing outcomes. The flexibility afforded by weekly measurement intervals will make it possible to disentangle bidirectionality across intervention components, malleable SRFOH, and health and well-being outcomes. This analysis will be accomplished using highly flexible methods for modeling discontinuous change [38].

#### Mediation analysis involving major measurements intervals

For mediation analyses involving the ultimate prevention outcomes, analyses will investigate how intervention delivery (dose, fidelity, components) impacts future changes in SRFOH, intermediate prevention outcomes, and ultimate prevention outcomes. Weekly intervention delivery measures will be aggregated across time (months 1–8; 10–17) to coincide with, yet precede, the assessment of mediators and outcomes in the major assessments. The mediators and outcomes will be measured at months 9 and 18, and models will adjust for baseline measurements of these variables. The statistical models will follow the above procedures, using an unconflated multilevel structural equation model approach.

#### Statistical power

There were two estimates: (1) the level of power to detect an effect for a *specific path* of the proposed mediation model and (2) the level of power to detect the *overall mediated effect*. The estimates assume *N* = 250 parents and are penalized for nesting within Coaches [39]. For the primary a-path models, with SRFOH as the direct target of Just Care for Families intervention components, power is 0.80 (α = 0.05) to detect a small-to-medium effect of *R*^2^ = 0.049. For the primary b-path models, where a change in SRFOH predicts intermediate or ultimate prevention outcomes, the detectable effect is *R*^2^ = 0.053. For the mediated effect, published tables [40] indicate that the proposed sample and measurement design are sufficient to detect a significant effect if the a-path has a medium effect and the b-path has a small-to-medium effect (or vice versa).

#### Aim 2. Examine how Just Care for Families program components are impacted by external structural SRFOH

This aim focuses on less malleable SRFOH, which are external, structural county-level attributes. With only five counties, these variables will be included at the upper level of the model (i.e., Coaches). Prior to analysis, the distribution of each variable will be evaluated to determine the appropriate scoring approach for modeling. The analyses will proceed in two steps. The first will be to evaluate the overall association between less malleable SRFOH and each model component from Aim 1 (i.e., Just Care for Families intervention components, individual/systemic SRFOH, and prevention outcomes), with a separate model for each component and SRFOH. The less malleable SRFOH variable will be entered as a main effect and interaction with the phase and/or time terms, indicating the extent to which change in malleable SRFOH differs for parents and Coaches in counties with different statuses on less malleable SRFOH. The second step will be to test for differential associations in each model path from Aim 1, that is, evidence of associations that vary as a function of less malleable SRFOH. As such, the paths detailed for Aim 1 will be extended to include less malleable SRFOH variables. The latter models will be formulated and interpreted using standard methods for moderated mediation [41, 42].

#### Aim 3. Examine how Just Care for Families program components can influence the relationship between SRFOH and individual/systemic outcomes on clinic-borne cost

System dynamics modeling will be used to evaluate and simulate Just Care for Families costs. This complexity-based approach is well-suited for analyzing a multi-level intervention, such as Just Care for Families, because it can effectively handle the non-linear, gradual nature of parent trajectories toward well-being and multi-directional relationships between risk factors [43, 44].

Economic analyses will take the systemic perspective, including clinic-borne (i.e., sites delivering Just Care for Families) and payer-borne (e.g., Medicaid) costs. Analyses will be completed in three steps using mixed methods [45].

*Step 1) Consolidate SRFOH data and generate parent profiles using Causal Loop Diagrams (CLDs)*. CLDs are qualitative system dynamics models that offer dynamic hypotheses of behaviors, including individuals’ behaviors [46]. The interconnections between behaviors and between effects and determinants of these behaviors create a system of factors or outcomes. Based on previous semi-structured interviews with Coaches and graduated parents [47] a CLD was developed to capture how individual, interpersonal, and structural SRFOH affect parental O/MU and MH symptoms. This CLD will be refined to reflect the experiences that parents and Coaches report during weekly digital check-ins. The refined CLD will inform the quantitative system dynamics model structure (step 2). The CLD also will be used to empirically derive parent profiles by mapping SRFOH data collected through weekly parent and Coach check-ins. Parent profiles will be distinguished by patterns of when SRFOHs are addressed (e.g., date a SRFOH milestone is achieved, sequencing of SRFOH). Two coders will identify potential profiles by reviewing data mapped to the CLD and reaching consensus on an initial profile set. This set will be pruned by removing profiles for which too few parents can be assigned (*n* < 10), resulting in confidentiality concerns. Further pruning will be completed in consultation with the CAB, using a scripted, consensus-driven approach for engaging community members with system dynamics to improve model validity [48]. Cost evaluations will be conducted for each parent profile and across profiles in step 3 [49].

**Fig. 3:**
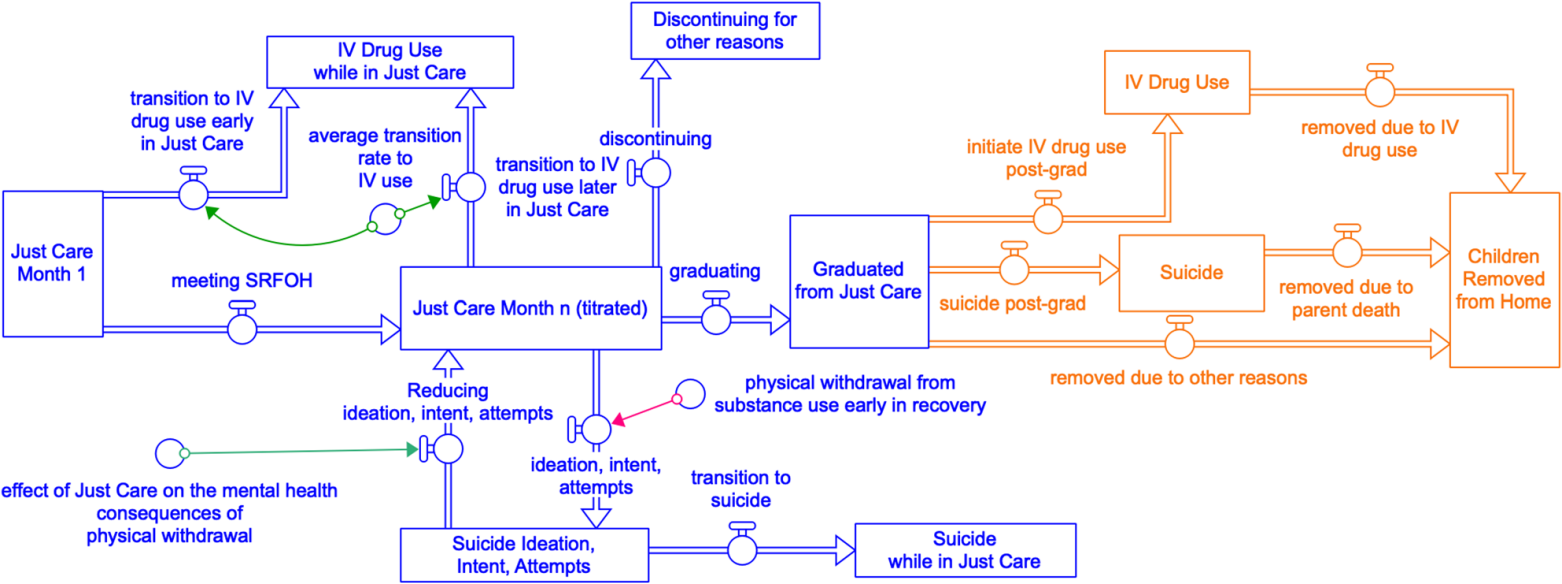
Core model structure for the simulation of Just Care for Families treatment trajectories

*Step 2) Develop simulation model structure*. Figure 3 displays a preliminary, core model structure. The structure will be refined using validation procedures described in Step 3. As currently conceptualized, parents will move through stocks (rectangles indicating variables tracked as aggregated values) as they titrate Just Care for Families sessions. Each stock will be associated with unbillable and billable Coach costs, as well as differential parent outcomes. Parents’ movements between stocks are represented by flows (arrows). Auxiliary variables (circles) alter flows. Critically, auxiliary variables model both individual-based inputs (e.g., withdrawal early in recovery; pink arrow Fig. 3) and intervention-based inputs (e.g., Just Care for Families engagement to help parents reduce suicidal ideation; green arrows Fig. 3). The values of stocks, flows, and auxiliary variables can vary by parent profile.

*Step 3) Evaluate observed costs and simulate potential future costs.* Costs will be evaluated by clinic, parent profile, and overall (i.e., full parent sample across the five clinics). Estimating costs by profile will help identify whether costs are substantially different based on which risk factors are addressed and the sequential ordering in which they are addressed [49]. The same model structure (step 2) can simulate clinic- or payer-borne costs. Further, the flexibility of simulation can estimate costs aligned with specific individual and structural level SRFOH. For example, clinics could estimate costs relative to the composition of their potential parent population (individual) or their county size, which would affect flexible costs such as mileage (structural/county).

The ingredients method [50] will be used to estimate indirect costs relative to program delivery that are both fixed (e.g., Coaches’ phones) and flexible, based on Just Care for Families dosage (e.g., mileage, engagement incentives). Costs specific to Just Care for Families start-up (e.g., Coach training time) will be estimated as a distinct ingredient category, as these costs are not directly impacted by the dynamic parent outcomes tracked in the simulation model.

The following costs will be reported: total cost to deliver Just Care for Families per parent, on average (i.e., fixed and flexible costs across parent profiles); clinic-borne cost to deliver Just Care for Families (i.e., difference between reimbursement and actual costs); total cost associated with treatment until each primary parent outcome (i.e., graduation, IV O/MU drug use, suicide); and weekly cost associated with key upstream milestones, such as the average cost to reduce parents’ suicidal ideation, intention, and attempts. Indirect and start-up costs will be added to the total cost and evenly distributed across outcome and parent-level costs. The model will use a one-week timestep, the shortest unit of time for which data will be available. The time horizon will be a minimum of 18-months, equivalent to the last major assessment time point. The model also will be run to include the cost for parents who terminate treatment prematurely due to either parent or Coach choice without achieving successful graduation.

Model validation will include structure assessment (i.e., does the structure reflect the real world?) [46] and surprise behavior (i.e., are there discrepancies between model behavior and the real world?) [46]. These will be completed with the Just Care for Families developer and Just Care for Families Coaches, given their expertise in Just Care for Families delivery. Behavior reproduction will entail calibrating baseline model estimates to clinic data from the proposed study period to demonstrate that real-world trends are simulated [46].

Minimum and maximum observed values will be used for one-way sensitivity analyses and probabilistic sensitivity analyses (PSA) over 10,000 Monte Carlo simulations [51, 52]. PSA parameter distributions will be selected following best practices (e.g., gamma for costs, beta for probabilities) [51, 52]. One-way sensitivity analyses will be conducted in Stella Architect and Microsoft Excel. PSA will be conducted in Crystal Ball [53].

The proposed model could be extended to other perspectives or to conduct different economic evaluations. For example, a benefit-cost analysis [45, 54, 55] could include costs important to parents (e.g., wages foregone while addressing other SRFOH) and monetize benefits or outcomes (e.g., days free from suicidal ideation, an additional year of education completed) [56] using well-established procedures [56]. The proposed analyses could also be extended into budget impact analyses for child welfare or other systemic perspectives by including costs associated with second-generation/child outcomes (e.g., foster care placements). Finally, analyses could consider the impact of providing Coach incentives for documentation for research purposes on clinic operations. Though not proposed as an implementation strategy for program delivery, this data collection strategy might demonstrate benefit to clinic efficiencies.

## Discussion

### Ongoing system engagement

This protocol will be conducted with ODHS serving as a key community partner. As noted, county offices will provide referrals and collaborate with Coaches to work with parents to set goals consistent with system recommendations. Further, state ODHS leadership will collaborate by supporting the program in the participating counties and encouraging sustainment. At a minimum, ODHS will be updated on study outcomes annually. If outcomes are promising, partner meetings will transition to planning for potential scale-up into other Oregon counties. These meetings will include policymakers and legislators to consider funding streams.

### Summary

This protocol describes a study of 250 parents enrolled in Just Care for Families in five rural Oregon counties. With a goal of examining the prevention of IV drug use and suicide in parents involved or at-risk for involvement with the CWS with an O/MU and MH disorder, this study will evaluate the impact of Just Care for Families on SRFOH and evaluate mechanisms through which Just Care for Families impacts prevention outcomes. Multi-component interventions like Just Care for Families have the potential to significantly change the SRFOH of families involved in child welfare, improving the long-term trajectory for well-being across generations, creating a significant public health impact.

## Data Availability

Data sharing is not applicable to this article as no datasets were generated or analyzed for this protocol. De-identified, non-administrative data collected for this trial will be deposited into the National Addiction and HIV Data Archive Program (NAHDAP).

## Abbreviations

SRFOH: Social Risk Factors of Health
O/MU: Opioid and/or Methamphetamine Use
CWS: Child Welfare System
IV: Intravenous
MH: Mental Health
ODHS: Oregon Department of Human Services
